# A new species of Bamboo (Poaceae, Bambusoideae) from coastal, southern Vietnam

**DOI:** 10.3897/phytokeys.272.188481

**Published:** 2026-03-24

**Authors:** Tran Van Tien

**Affiliations:** 1 Faculty of Biology, Dalat University, 01 Phu Dong Thien Vuong, Dalat, Lam Dong, Vietnam Faculty of Biology, Dalat University Dalat Vietnam

**Keywords:** Bambusoideae, endemism, morphology, new taxon

## Abstract

A newly discovered species of the paleotropical woody bamboo subtribe Bambusinae (Poaceae, Bambusoideae), *Cochinchinochloa salta* V.T. Tran, from coastal areas of Phu Yen Province, South-Central Vietnam, is described and illustrated. Based on vegetative and reproductive characters, *Cochinchinochloa salta* shares characters with *Cochinchinochloa braiana*, but differs from it by having nodes of leafy branches with a thin swollen patella, bud elliptic, the culm leaf blade base slightly protruding, auricles curved and small, and nucoid caryopsis oblique with a relatively thick pericarp.

## Introduction

The subtribe Bambusinae (Bambuseae) comprised 16 genera, five of which are clambering or scrambling bamboos distinguished by iterauctant inflorescences (Soreng et al. 2022). This group has attracted sustained taxonomic scrutiny over the past several decades (Wong 1993, 2005; Widjaja 1997; Nguyen and Tran 2013). Currently, five clambering or scrambling bamboo genera are recognized in Asia: *Maclurochloa* K. M. Wong, *Soejatmia* K. M. Wong from the Malay Peninsula (Wong 1993); *Melocalamus* Bentham & Hooker, distributed in Asia (Nguyen and Tran 2010); and the Vietnamese genera *Cochinchinochloa* (Nguyen and Tran 2013) and *Yersinochloa* (Nguyen and Tran 2016). The systematic delimitation of these genera has traditionally relied on a combination of vegetative and productive characters (Wong 1993, 2005; Widjaja 1997; Nguyen and Tran 2013, 2016), enabling the use of stable, diagnostically informative traits in taxonomic descriptions.

**Figure 1.**
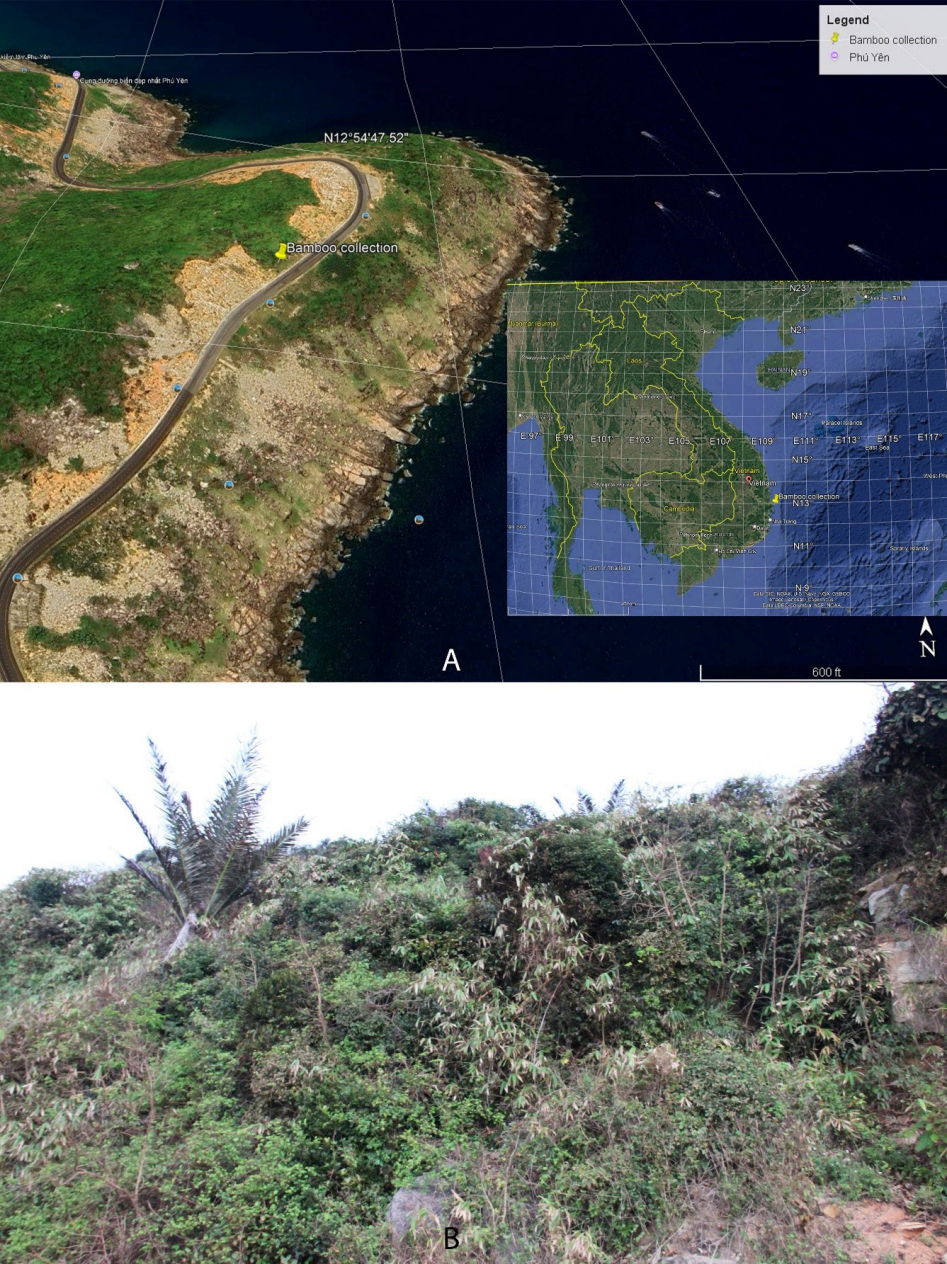
The habitat of *Cochinchinochloa salta* V.T.Tran. **A**. Location; **B**. Hábitat.

**Figure 2.**
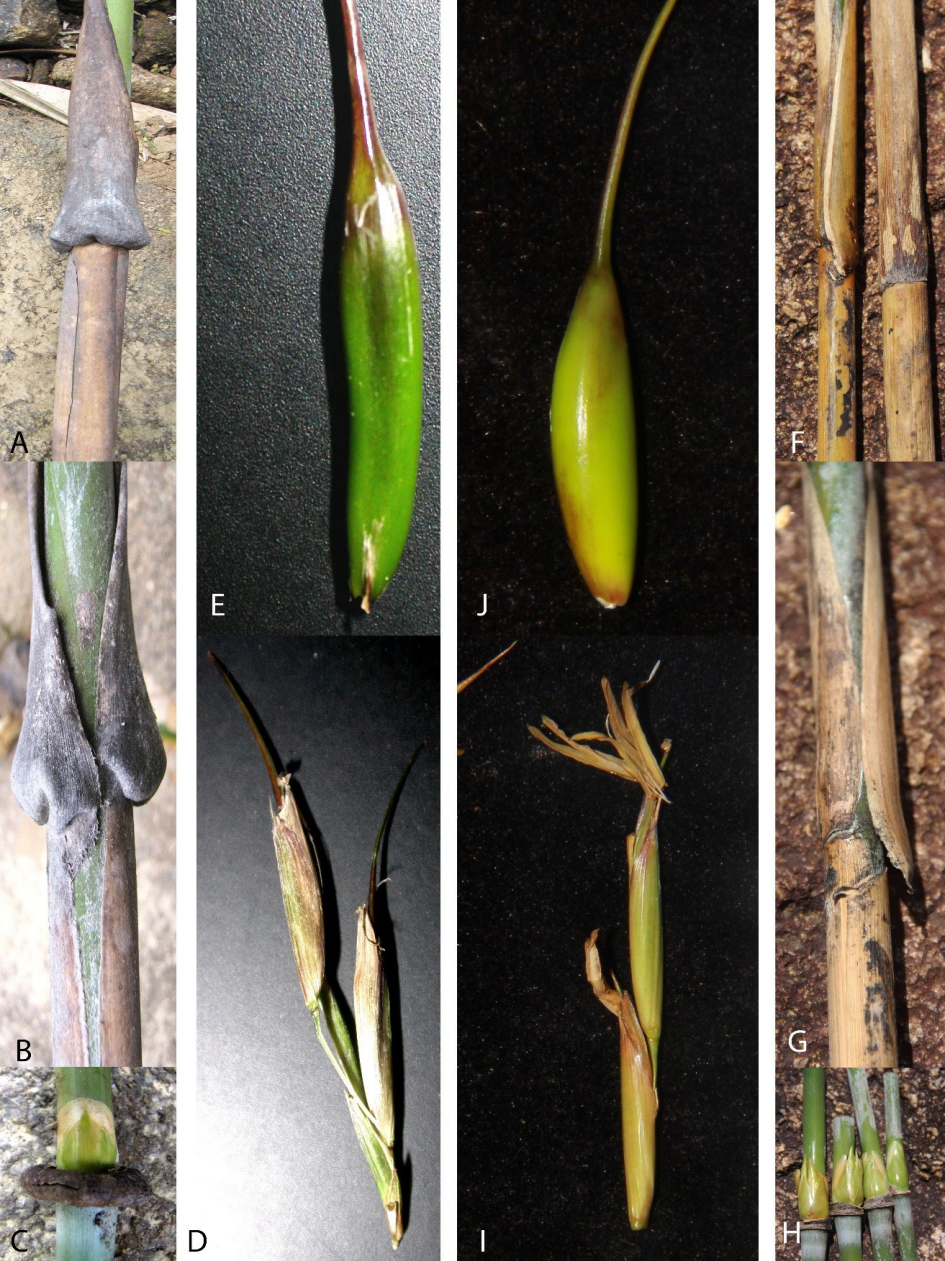
*Cochinchinochloa braiana* H.N.Nguyen & V.T.Tran (left), *C. salta* V.T.Tran (right). **A, B**. Culm leaves; **C**. Node and bud; **D**. Pseudospikelets; **E**. Caryopsis; **F, G**. Culm leaves; **H**. Nodes and bud; **I**. Pseudospikelets; **J**. Caryopsis.

*Cochinchinochloa* H.N.Nguyen & V.T.Tran, comprised 1 species, was described in 2013 (H.N.Nguyen et al. 2013), which is characterized by each node giving rise to several branches, with one becoming longer and dominant, a thick swollen patella at the culm nodes and nodes of leafy branches, pseudospikelets having two perfect florets, the rachilla internode between the perfect florets elongated, a rachilla extension bearing an imperfect floret at maturity. The species is distributed in evergreen broadleaf forests or degraded forests in high mountainous regions from southern Vietnam (Nguyen et al. 2013). During a series of collecting trips along coastal and island foredunes in Mui Dien, Hoa Tam Commune, Dong Hoa District, Phu Yen Province, South-Central Vietnam (Fig. 1) from 2020 to 2022, I conducted extensive field surveys and found several populations of a clambering bamboo that was widespread and abundant in heavily degraded natural scrub distributed on sloping stony coasts. Specimens of branches and flowering branches were collected, and detailed morphological observations were made. It clearly belongs to *Cochinchinochloa*, characterized by its culm nodes developing a patella; the inflorescences are borne terminally on leafy branches; in mature pseudospikelets the elongated rachilla internode bears a fertile floret which easily disarticulates at maturity; a rachilla extension is present, bearing an imperfect floret elongate at maturity; and the caryopsis is oblong with a relatively thin pericarp (Nguyen et al. 2013). However, its culms and branches are small with nodes having a thin swollen girdle, bud elliptic, the culm leaf blade base slight protruding, auricles curved and small, caryopsis oblique with a relatively thick pericarp, a unique combination of characteristics not found in any known species of the genus (Fig. 2). Therefore, the new collection of bamboo is formally described and illustrated below.

## Materials and methods

This study was conducted using plant material collected from Mui Dien in Hoa Tam commune, Dong Hoa District, Phu Yen Province, located in South-Central Vietnam. The plant specimens were deposited at the Herbarium of Dalat University (**DLU**) and the Tay Nguyen Institute for Scientific Research (**VTN**). Measurements of the vegetative parts were taken in the field, and color photographs were captured using a Canon 600D camera. Pseudospikelets were dissected using a Meiji Techno EM-32 stereomicroscope. A putative comparison was made with a presumably related species, *Cochinchinochloa braiana* H.N.Nguyen & V.T.Tran, to too strong the accuracy of our findings was based on plant material collected from Mui Dien, Hoa Tam commune, Dong Hoa District, Phu Yen Province, South-Center Vietnam.

### Taxonomic treatment

#### Cochinchinochloa salta

Taxon classificationPlantaePoalesPoaceae

V.T.Tran sp. nov.

FC7ADCAE-0DAD-5A9E-AA9B-7477A1EB4F74

urn:lsid:ipni.org:names:77378055-1

Fig. 3

##### Type.

Vietnam • Phu Yen Province: Dong Hoa District, Hoa Tam commune, Mui Dien degraded forests, stone slopes, along coastal, 12.54378, 109.27105, 06 October 2025, *Tran Van Tien, TVT-DLU 0465* (holotype: DLU; isotypes: VTN!).

**Figure 3.**
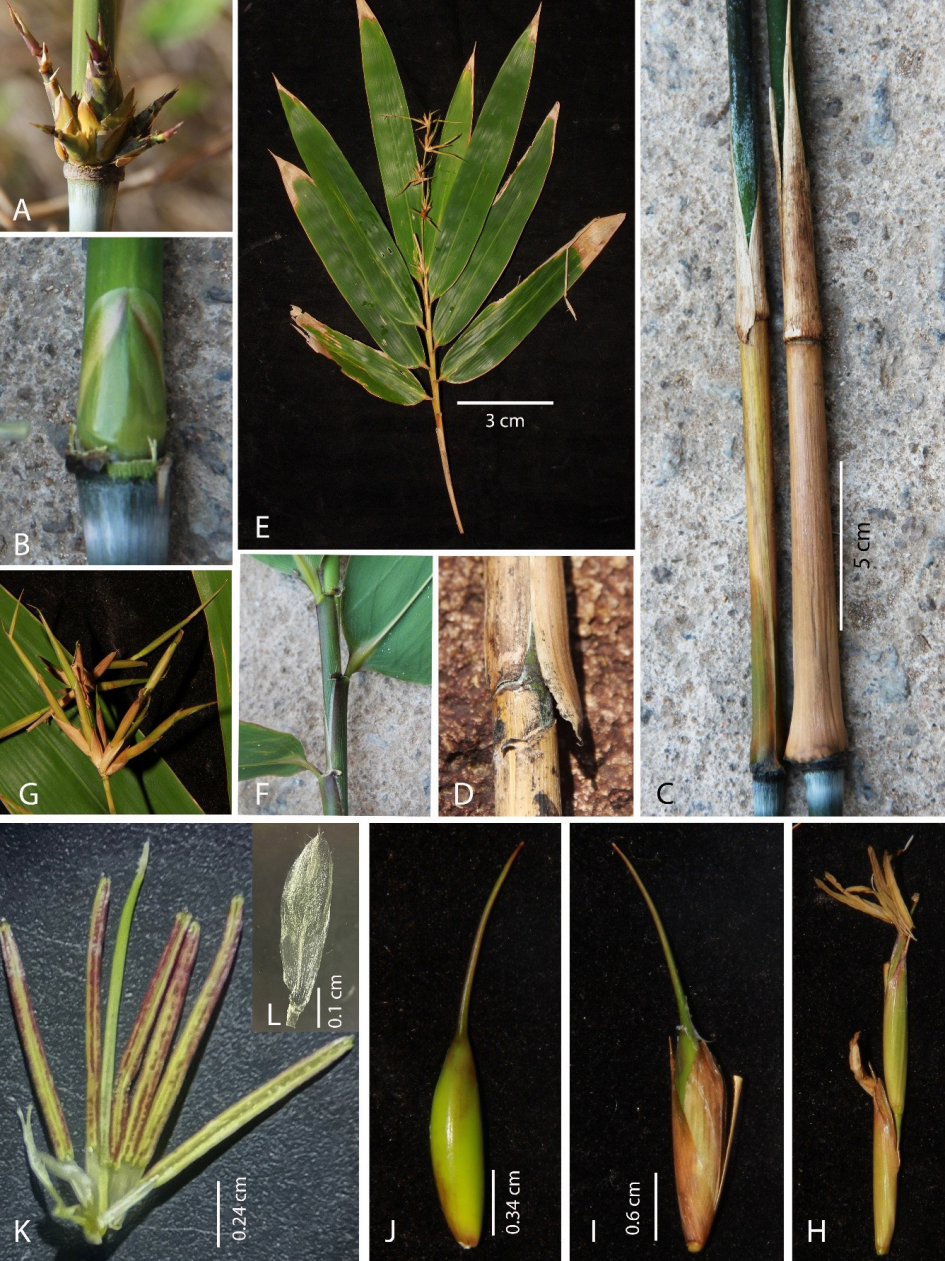
*Cochinchinochloa salta*. **A**. Section of culm with 4–6 branches and middle one dominant; **B**. Bud and node; **C**. Culm leaves; **D**. Section of culm leaf with small auricles; **E**. Leafy branches. **F**. Section of leafy branch with auricles. **G**. Inflorescence. **H**. Pseudospikelets; **I**. Floret; **J**. Caryopsis; **K**. Stamens; **L**. Lodicule. Photo by. V.T. Tran from specimens used for preparation of the holotype.

##### Diagnosis.

*Cochinchinochloa salta* is distinguished from *C. braiana* by culm-leaves hairs inconspicuous (vs culm-leaves hairs); nodes thin, a swollen girdle (vs. a thick swollen girdle); bud elliptic (vs. bud ovate), the culm leaf blade base slight protruding (vs. the blade base protruding); auricles curved, 0.1 × 0.1 cm (vs. triangle shaped, 0.6–0.8 × 1.5–2.5 cm), abaxial lemma and palea smooth, veins inconspicuous (vs. cilia, veins prominent); caryopsis oblique with a relatively thick pericarp (vs. with a relatively thick pericarp)

##### Description.

Culms young erect, and then scrambling or hanging over nearby vegetation at maturity, 3–5 m tall; internodes 30–40 cm long and 0.5–1 cm in diameter, when young sparsely covered with appressed white hairs; culm walls thick, 0.3–0.7 cm; nodes with a thin swollen girdle, bud elliptic. Culm leaves yellow green when young, hairs inconspicuous; 15–20 cm long and 0.7–1.2 cm wide, the blade base slightly protruding, margins bearing inconspicuous hairs; blade tardily deciduous, erect, ca. 1 cm long, hairs inconspicuous; auricle 0.1 × 0.1 cm; ligule very short, entire. Branches 4–6 with middle one dominant, elongating. Leafy branches 40–60 cm long, bearing 5–8 leaves; blades oblong-obovate, base broadly rounded, glabrous, 4.5–5.0 × 28–31 cm, veins in 10–12 pairs; petiole 1.2–1.3 cm long; sheaths bearing curve auricles c. 0.1 × c. 0.2 cm with dense fimbriae, 2–3 mm long; inner ligule a low rim, c. 1 mm; pseudopetiole ca. 0.2 × ca. 0.2 mm. Inflorescences terminating leafy branches, indeterminate; pseudospikelets typically 3–4 cm long when young and 5–6 cm long at maturity, each subtended by a prophyllate bud and consisting of 1–3 transitional glumes, having 2–4 perfect florets and a vestigial terminal flower. Rachilla internode below the lower fertile floret does not elongate at maturity, ca. 0.2 cm; rachilla internode between perfect floret elongate, ca. 1.3 cm. Perfect florets 1.6–1.8 cm; lemmas oblong-lanceolate, abaxial smooth, 0.3–0.4 × 1.3–1.4 cm, veins inconspicuous, 10–12, apex mucronate, mucro 1–2 mm long, margins bearing dense white cilia at the apex; palea 2-keeled with a narrow groove on the back, abaxial smooth, 0.2–0.3 × 1.2–1.4 mm, veins inconspicuous, apex bifid, margins bearing white cilia at the top; lodicules 3, obovate, 0.1 × 0.4–0.5 cm, margins ciliate upper; stamens 6, filaments free, 0.3–0.4 × 1.2–1.3 cm, apices bearing tiny spines, ca. 0.5 mm; ovary glabrous with a long style and stigmas 3; nucoid caryopsis green when fresh, oblique with a relatively thick pericarp, 1.5–1.7 cm long.

##### Distribution and ecology.

*Cochinchinochloa salta* is currently known only from the type locality in Hoa Tam Commune, Dong Hoa District, Phu Yen Province, South-Center Vietnam. It grows in remnants of degraded natural scrub, on stone slopes, and along the coast.

##### Etymology.

The specific epithet salta refers to bamboo that grows along the coast and is influenced by sea salt.

##### Phenology.

The plants were flowering from August to October 2025. New shoots are developed from August to October.

##### Preliminary conservation status.

An extensive survey in the thousand-hectare area revealed only known from a single population in Hoa Tam Commune, Dong Hoa District, Phu Yen Province, South-Center Vietnam. This population has no more than 500 mature clumps, all growing in heavily degraded natural scrub. According to the IUCN Red List Categories and Criteria (IUCN 2022), this species is classified as Data Deficient (DD) and requires further surveys.

## Discussion

Several comprehensive studies on bamboos have demonstrated that careful investigation of vegetative characters can provide sufficient diagnostic features for species identification (Holttum 1958; Wong 1995). Morphological traits have been identified for comparison among different bamboo species. These traits include the shape of the culm bud and the presence of indumentum on the internodes, as seen in *Ampelocalamus sinovietnamensis* (Y.H. Tong, Z.G. Xu, J.B. Ni & N.H. Xia, 2021). Other noteworthy characteristics are the glabrous nodes and culm leaf sheaths found in *Chimonocalamus elegans* (Sungkaew & Teerawat, 2017), as well as the presence and shape of auricles in *Chimonocalamus auriculatus* (Sungkaew, Hodk. & N.H. Xia, 2018). Additionally, morphological differences in the internode buds and culm sheaths have been noted among various species of the genus *Yersinochloa* (Tran et al. 2023; Duy et al. 2024). Morphological analysis (Table 1, Fig. 2) shows that the unknown bamboo differs from the closely related species in several vegetative and reproductive characters.

**Table 1.** Morphological comparisons of of *Cochinchinochloa salta* V.T.Tran, sp. nov. with *C. braiana* H.N.Nguyen & V.T.Tran.

Characters		*C. salta*	*C. braiana*
Internodes	internodes	0.5–1 × 30–40 cm	2–3.5 × 60–80 cm
	node	thin swollen girdle	thick swollen girdle
	bud	elliptic	ovate
Culm leaves	culm-leaves blade	the blade base slight protruding	the blade base protruding
	hairs	hairs inconspicuous	densely appressed white hairs
	auricles	curved, 0.1 × 0.1 cm	triangle shaped, 0.6–0.8 × 1.5–2.5 cm
Perfect fertile florets		2–4	2–3
		abaxial lemma smooth, veins inconspicuous	abaxial lemma cilia, veins prominent
		abaxial palea smooth, veins inconspicuous	abaxial palea cilia, veins prominent
Caryopsis		oblique with a relatively thick pericarp	oblique with a relatively thin pericarp

## Supplementary Material

XML Treatment for Cochinchinochloa salta

## Additional information

### Use of AI

No use of AI was reported.

### Author ORCIDs

Tran Van Tien https://orcid.org/0000-0001-6539-0327

### Data availability

All of the data that support the findings of this study are available in the main text.
